# DFusMol: predicting molecular properties based on dual-channel attention

**DOI:** 10.3389/fmolb.2025.1623620

**Published:** 2025-07-30

**Authors:** Xuan Liu, Wei Du, Haibao Tang, Yingjian Gu, Zhibang Li, Xiaoyang Fu

**Affiliations:** ^1^ College of Computer Science and Technology, Jilin University, Changchun, China; ^2^ School of Computer Science, Zhuhai College of Science and Technology, Zhuhai, China; ^3^ School of Life Sciences, Jilin University, Changchun, China

**Keywords:** multi-modality learning, deep learning, molecular property prediction, graph neural networks, transformer, molecular graphs

## Abstract

Accurate molecular property prediction is fundamental to modern drug discovery and materials design. However, prevailing computational methods are often insufficient, as they rely on single-granularity structural representations that fail to capture the hierarchical complexity of molecular systems. To address this challenge, we propose a new approach to molecular representation learning that incorporates structural information across multiple scales. We design DFusMol (Dual Fusion with Global and Local Attention), a novel framework inspired by multi-modal learning. DFusMol employs graph encoders to capture features from both atomic-level molecular graphs and motif-level graphs derived from chemical rules. A customized global-local attention mechanism then blends these diverse features to build comprehensive molecular representations. Experiments on nine public benchmark datasets reveal that DFusMol delivers top-tier predictive performance across all tasks, outperforming state-of-the-art self-supervised learning models on six of them. By effectively integrating atomic- and motif-level information, DFusMol provides an innovative and efficient solution for molecular property prediction, enhancing representation learning methodologies and demonstrating strong potential for applications in drug design and lead compound screening.

## 1 Introduction

The prediction of molecular properties is a fundamental task in drug discovery, materials science, and environmental studies (Fedik et al., 2022). In the domain of drug discovery, the development pipeline is notoriously lengthy and complex, characterized by substantial costs and high attrition rates (Hay et al., 2014; Dowden and Munro, 2019). Therefore, the ability to computationally predict a molecule’s physicochemical properties, biological activities, and toxicity *a priori* is of great importance (Hou et al., 2022; Yang et al., 2025). Such predictive models can significantly reduce experimental expenditures, accelerate the identification and optimization of promising lead compounds, and provide valuable guidance for rational molecular design (Sadybekov and Katritch, 2023).

Early approaches to molecular property prediction predominantly relied on expert-defined rules and classical machine learning models (Deng et al., 2023). In these methods, molecules were typically represented by manually engineered features, such as molecular descriptors (Harnik and Milo, 2024), or structural representations like molecular fingerprints (Keiser et al., 2009) and SMILES strings (Weininger et al., 1989). These features were then used as input for algorithms like Support Vector Machines (SVM) (Boser et al., 1992) and Random Forests (RF) (Breiman, 2001) to perform the prediction task (Wei et al., 2016; Ahneman et al., 2018). While these pioneering efforts established a foundation for the field, they suffered from significant limitations. A primary drawback was their difficulty in elucidating the complex topological information inherent in molecular structures (Deng et al., 2023). Furthermore, the reliance on manually engineered features limited their generalizability and adaptability to a diverse range of molecular property prediction tasks (Zeng et al., 2022).

With the rapid advancement of deep learning, researchers began to represent molecules as graph structures, where atoms are treated as nodes and chemical bonds as edges. This paradigm has established Graph Neural Networks (GNNs) as a cornerstone of modern molecular modeling (Guo et al., 2022; Zhang et al., 2023). Libraries such as RDKit (Landrum, 2016) have greatly facilitated this graph-based representation. For example, Gilmer et al. introduced the Message Passing Neural Network (MPNN) (Gilmer et al., 2020), which dynamically updates node features through a message-passing mechanism. Subsequent works, including DMPNN (Gasteiger et al., 2020) and CMPNN (Song et al., 2020), further refined this approach by incorporating the joint influence of edges and nodes, thereby enhancing the model’s capacity to discern intramolecular interactions and electronic effects. A key advantage of these GNN-based methods over traditional approaches is their ability to learn features automatically, eliminating the need for manual feature engineering. However, early GNN models were often limited by their reliance on local neighborhood information. To address this, recent research has focused on more sophisticated molecular representation learning. MetaGIN (Zhang et al., 2025) introduced a 3-hop convolution to encode 3D structural information such as bond lengths, angles, and dihedral angles. The Frad framework (Ni et al., 2024) employed carefully designed noise to simulate molecular vibrations and rotations. Furthermore, Li et al. proposed an automated graph learning method (Li et al., 2022) that reduces manual intervention and significantly improves molecular representation fidelity. These advancements have created new opportunities in virtual screening and materials design, yet their predominant focus on local information can still impede the effective propagation of information between distant parts of a molecule, thus limiting their ability to fully characterize global molecular structures and properties.

The emergence of the Transformer model (Ashish, 2017) in natural language processing has provided new perspectives for molecular modeling. Originally introduced by Vaswani et al., the Transformer architecture utilizes a self-attention mechanism to model long-range dependencies between sequence elements, achieving state-of-the-art results in tasks like machine translation. The operational principles of self-attention bear a notable resemblance to those of Graph Attention Networks (GAT) (Veličković et al., 2017), as both are adept at dynamically aggregating global information. Inspired by this connection, Schwaller et al. pioneered the application of Transformers to molecular property prediction with their Molecular Transformer model (Schwaller et al., 2019). This model represents molecules as SMILES strings and employs a Transformer encoder to extract meaningful features, demonstrating performance comparable to leading GNN-based models. This success highlighted the potential of Transformers in this domain and spurred a series of studies fusing Transformer architectures with GNNs (Sultan et al., 2024). Building on this trend, Rong et al. developed GROVER (Rong et al., 2020), which encodes molecular substructures using graph isomorphism networks and processes the entire molecular graph with a self-attention mechanism. MoLFormer (Ross et al., 2022) utilizes a Transformer encoder enhanced with rotational positional encoding and leverages unsupervised pre-training on large-scale SMILES datasets to generate informative molecular embeddings. Zhou et al. combined Transformer designs with SE (3) transformations to create UniMol (Zhou et al., 2023), a versatile 3D pre-training framework capable of both representing and predicting molecular 3D conformations. Similarly, TransFoxMol (Gao et al., 2023) incorporates chemistry-informed spectrograms to refine its attention mechanism, effectively unifying global and local molecular information.

In summary, the integration of Transformer models has significantly advanced the field of molecular representation. These models provide a powerful framework for capturing global molecular semantics and often serve to complement the local feature extraction capabilities of Graph Neural Networks (GNNs). By synergistically combining the strengths of both architectures, these hybrid approaches have expanded the scope and improved the predictive accuracy for a wide array of molecular property prediction tasks.

Despite these advancements, a reliance solely on atomic-level information, even within sophisticated GNN and Transformer frameworks, presents a notable limitation. It is well-established that the arrangement and interplay of functional substructures (i.e., chemical motifs) within a molecule profoundly influence its chemical properties and biological activities. For instance, substructures like aromatic rings are known to contribute to molecular stability and lipophilicity, while functional groups such as amino and carboxyl moieties dictate properties like acidity, basicity, and reactivity—attributes that are critical determinants of a drug’s solubility and bioavailability. Therefore, the incorporation of this higher-level structural information is essential for developing more comprehensive and accurate predictive models (Hall et al., 1991).

Addressing this need, several studies have explored motif-based representations. Zhang et al. introduced MGSSL (Zhang et al., 2021), a self-supervised learning model that enhances GNNs by capturing information from functional groups and motifs. Similarly, Fang et al. developed a method that utilizes functional group-augmented graphs within a contrastive pre-training framework based on chemical knowledge graphs (9). However, these existing methods exhibit limitations in how they merge information across different structural levels. Firstly, the fusion strategy between the atomic-level molecular graph and the higher-level motif graph is often simplistic, failing to fully delineate the complex semantic relationships between these multi-scale features. Secondly, they typically lack a flexible mechanism to effectively weigh and combine local features (derived from motifs) with global features (representing the overall molecular topology). These shortcomings can impede model performance, particularly for complex property prediction tasks where such hierarchical interactions are crucial.

To address the aforementioned limitations, we propose a novel molecular representation framework named DFusMol (Dual-Channel Attention for Graph Fusion), which systematically integrates information from both molecular graphs and their corresponding motif graphs. Within this framework, we utilize the Communicative Message Passing Neural Network (CMPNN) (Song et al., 2020) as the graph encoder to generate high-quality atomic embeddings through multi-layer message passing between nodes and edges. Concurrently, we employ a molecular decomposition algorithm grounded in chemical principles to construct a motif-level graph. This graph explicitly records higher-level substructural features, including ring systems and functional groups, as well as their contextual interconnections. A core contribution of our work is a dual-channel attention mechanism designed for hierarchical graph fusion. By leveraging adjacency and distance matrices, we have designed two distinct attention channels: a local channel and a global channel. The local attention channel is engineered to precisely pinpoint fine-grained interactions between individual motifs and their immediate chemical environment. In contrast, the global attention channel focuses on modeling the relationships between these motifs and the overall molecular topology. This dual-channel strategy enables DFusMol to comprehensively aggregate molecular structural information from diverse perspectives (Zhang et al., 2023), thereby achieving a more profound and detailed understanding of molecular structures.

## 2 Methods

**FIGURE 1:**
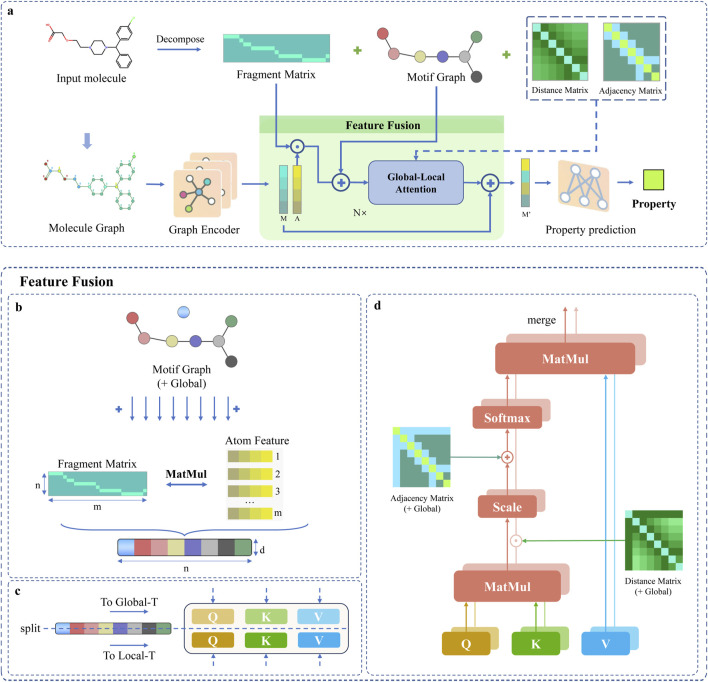
Overview of the DFusMol Framework: **(a)** Presents the overall architecture of the model, where a decomposition algorithm is first utilized to extract the motif structures and features of the molecule, a graph encoder captures the interactions between nodes and edges, and comprehensive feature fusion is conducted in the Feature Fusion module, followed by precise analysis via Global-Local Attention. **(b)** Describes the fusion process between motif-level and atomic-level features, as detailed in the “Dual-Channel Feature Fusion” section. **(c)** Details the segmentation of the fused graph. **(d)** Elaborates on the core components of the dual attention mechanism, as detailed in the ‘Global-Local Attention’ section.

The DFusMol framework is depicted in Figure 1a, with its upper part showing the motif decomposition guided by chemical principles and its lower part illustrating the molecular feature fusion based on the attention mechanism.

### 2.1 Motif-based structural representation

**FIGURE 2:**
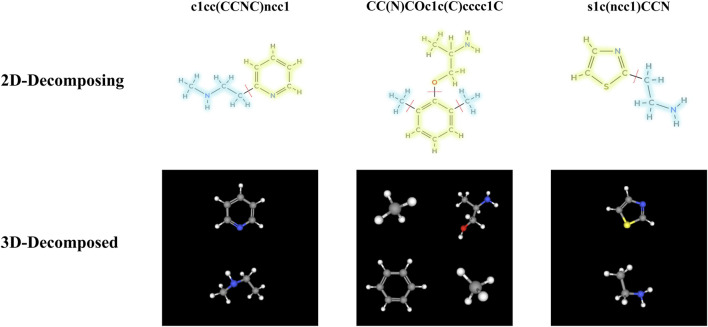
Decomposition of three randomly selected molecules into motif fragments using the molecular fragmentation function.

Our study is centered on a graph transformation framework that utilizes chemical motifs to effectively encode the chemical semantics of molecules. Specifically, we adopt a molecular fragmentation approach informed by established chemical principles (Zhang et al., 2021; Zhong et al., 2024). This approach partitions molecular graphs into motif fragments according to two primary rules: (Ahneman et al., 2018): Cleavage of bonds connecting ring systems to substituents; and (Ashish, 2017) Identification of non-ring atoms with three or more bonded neighbors as distinct motifs. Application of these rules to the ChEMBL (Gaulton et al., 2012) database yielded a vocabulary of 12,331 unique motifs. For illustrative purposes, three molecules were randomly selected from the BBBP dataset within the MoleculeNet (Wu et al., 2018) benchmark, and their detailed bond-breaking steps are shown in Figure 2.

Following the decomposition rules, each molecule is converted into a motif graph G=(V,E) , where V is the set of motif nodes and E represents the connections between motifs that share atoms. For each motif i∈V , we construct a feature vector $M_{i}\in R^{L}$ , where L is the size of the motif vocabulary. This vector is a one-hot encoding, where the element corresponding to the motif’s index in the vocabulary is assigned a value of 1, while all other elements are 0 (Yu and Gao, 2022). We also incorporate two special nodes: a [GLOBAL] node to aggregate graph-level information, and an [UNK] node to represent motifs not present in the vocabulary. The distance between the [GLOBAL] node and all other motif nodes is defined as 1.

To fully represent the structural properties of the motif graph, we construct three essential auxiliary matrices:

$$A\in {\left\{0,1\right\}}^{\left(n+1\right)\times \left(n+1\right)},\;D\in N^{\left(n+1\right)\times \left(n+1\right)},\;F\in {\left\{0,1\right\}}^{n\times m}$$

• The adjacency matrix A encodes the direct connectivity between motifs.

• The distance matrix D stores the shortest path lengths between all pairs of motifs.

• The motif-atom association matrix F specifies the membership of atoms within each motif; if motif i contains atom j , then $F_{ij}=1$ , otherwise $F_{ij}=0$ .

Note that F is generated based on the initial molecular decomposition and excludes the special nodes. The resulting matrix F has dimensions n×m , where n is the number of motifs and m is the total number of atoms in the molecule. In contrast, A and D are constructed after the inclusion of the special [GLOBAL] node, resulting in dimensions of (n+1)×(n+1) .The adjacency matrix encodes local structural topology and provides neighborhood information, while the distance matrix helps the model understand the overall size, branching structure, and long-range relationships of the molecular graph. In high-performing models, both matrices are indispensable.

### 2.2 Construction of molecular graphs

In our framework, a molecule is formally represented as a directed graph G=(V,E) . The set of nodes, $V=\left\{v_{1},v_{2},\ldots ,v_{\left|V\right|}\right\}$ , corresponds to the atoms in the molecule, while the set of edges, E={e(u,v)∣u,v∈V} , represents the chemical bonds. Each edge e(u,v) is directed, signifying a bond from atom u to atom v .

Each node v∈V is initialized with a feature vector $h_{v}^{0}\in R^{d}$ , where d is the dimensionality of the atomic features. These features comprise the atom’s chemical attributes (e.g., type, aromaticity, mass), as well as its path position and 3D spatial distance information.

Similarly, each directed edge e(u,v)∈E is associated with a feature vector $h_{uv}\in R^{b}$ , where b is the dimensionality of the bond features. These features include properties such as (Ahneman et al., 2018) bond type (e.g., single, double, or aromatic) and (Ashish, 2017) conjugation status and other related chemical attributes.

The choice of a directed graph representation over an undirected one is a deliberate design decision to enhance the model’s representational power. Directed graphs are better suited to capture the directional nature of chemical bonds, a property that is crucial for determining many molecular properties. This is particularly salient for chiral molecules, where stereochemistry is a pivotal determinant of biological activity.

### 2.3 Graph encoder

In our model, we construct the graph encoder based on the Communicative Message Passing Neural Network (CMPNN) (Song et al., 2020). We chose CMPNN as its core message-passing scheme is inherently built on directed graphs, directly addressing our need to model bond directionality. Furthermore, it is a lightweight architecture that converges quickly without requiring pre-training. The encoder operates in three key steps: aggregating messages from edges to nodes, updating node features to refine edge features, and concluding with a final message aggregation.

Initially, at each layer k of message passing, every node v aggregates messages from its incoming edges e(u,v) and combines them to form an aggregated message $m_{v}^{\left(k\right)}$ :

$$m_{v}^{\left(k\right)}=\text{SUM}\left(\left\{h_{uv}^{\left(k-1\right)}|e\left(u,v\right)\in E\right\}\right)\cdot \text{MAX}\left(\left\{h_{uv}^{\left(k-1\right)}|e\left(u,v\right)\in E\right\}\right).$$ (1)

Here, $h_{uv}^{\left(k\right)}$ represents the edge feature at layer k , SUM adds up all incoming edge features, and MAX picks out the edge feature with the strongest information. This combination captures both the overall information from the local neighborhood edges and their most salient features. Subsequently, the node updates its feature by combining the aggregated message with its current feature:

$$h_{v}^{\left(k\right)}=h_{v}^{\left(k-1\right)}+m_{v}^{\left(k\right)}.$$ (2)

This design ensures that nodes integrate local information from their neighborhood while preserving their own feature representations.

For each edge e(u,v) , its message is determined jointly by the feature of the source node u and the feature of the reverse edge e(v,u) . Specifically, the edge message is computed as the difference between the source node feature and the reverse edge feature:

$$m_{uv}^{\left(k\right)}=h_{u}^{\left(k\right)}-h_{vu}^{\left(k-1\right)}.$$ (3)

This process serves to modulate the direction of information flow. We then update the edge’s hidden state by applying a linear transformation and a nonlinear activation:

$$h_{uv}^{\left(k\right)}=\sigma \left(W_{k}\cdot m_{uv}^{\left(k\right)}+h_{uv}^{\left(0\right)}\right),$$ (4)

Here, σ refers to a nonlinear activation function, and $W_{k}$ is a learnable weight matrix. This update approach allows edge features to adapt to changes in node features, facilitating the propagation of more detailed information in later layers.

After completing message passing across all layers, the node features $h_{v}^{\left(k\right)}$ undergo an additional update by aggregating the current edge features and initial node features:

$$h_{v}=\text{COMMUNICATE}\left(h_{v}^{\left(k\right)}+m_{v}^{\left(k\right)}+x_{v}\right).$$ (5)

Finally, to generate the graph-level representation, we employ a sophisticated readout mechanism based on a bidirectional Gated Recurrent Unit (GRU) (Cho et al., 2014). Unlike simple permutation-invariant pooling functions (e.g., mean/sum) that treat node features independently, the GRU readout treats the set of node features in a graph as a pseudo-sequence and performs a learned, context-aware aggregation. The bidirectional nature of the GRU allows the final representation of each node to be informed by the global context of all other nodes in the graph. This process captures higher-order, non-local interactions between node features, producing a refined and highly expressive foundational representation of the entire molecule for the final prediction task.

For the atomic features obtained after message passing, a global molecular feature representation can be derived via average pooling. However, since molecules vary in their number of atoms, direct batch-wise fusion with the motif matrices is infeasible. To address this issue, we have designed an adaptation strategy. For a molecule i in a batch containing $m_{i}$ atoms, its atomic features are represented as a matrix $X_{i}\in R^{m_{i}\times d}$ . To handle the variable number of atoms across molecules within a batch, we introduce a padding operation:

$$X_{i}^{\text{pad}}=\text{Pad}\left(X_{i},M_{\text{max}}\right)\in R^{M_{\text{max}}\times d},$$ (6)

$$X_{i}^{\text{pad}}\left[j\right]=\left\{\begin{array}{cc} X_{i}\left[j\right]\; & \text{if }j<m_{i}\\ 0\; & \text{if }m_{i}\leq j<M_{\text{max}} \end{array}\right.,$$ (7)

where $M_{\text{max}}=\max\left(m_{i}\right)$ denotes the maximum number of atoms in the batch, and the padding operation appends zero-vector rows to the matrix $X_{i}$ until it reaches a dimension of $M_{\text{max}}\times d$ . In this context, $X_{i}\left[j\right]$ represents the feature vector of the j -th atom in molecule i .

### 2.4 Dual-channel feature fusion

A core innovation of DFusMol is its hierarchical feature fusion mechanism, designed to create a unified representation that integrates fine-grained atomic features with high-level chemical motif semantics. This process transforms atomic-level information into motif-level representations, which are then fused with the motifs’ own identity embeddings. The fusion is performed in three distinct steps, as detailed below.

The initial step is to project the atomic features onto the motif graph. We leverage the motif-atom association matrix $F_{i}\in R^{n\times m}$ as a linear operator to aggregate atomic features. The matrix multiplication $F_{i}X_{i}$ effectively sums the feature vectors of all atoms belonging to each respective motif. This operation produces an intermediate matrix where each row vector represents the collected atomic information for a single motif.

A simple summation, however, can bias the representation towards motifs composed of a larger number of atoms. To mitigate this, we introduce a normalization factor. We then apply a learnable linear transformation to project the normalized, aggregated features into the target embedding space of dimension $d^{\prime }$ . This step is crucial for dimensional alignment and for increasing the model’s expressive capacity. The combined operation is:

$$H_{i}^{\text{atomic\_proj}}=\text{ReLU}\left(\frac{1}{m}F_{i}X_{i}W_{G}+b_{G}\right)$$ (8)

where $\frac{1}{m}$ serves as a simple and effective normalization term, with m being the total number of atoms. $W_{G}\in R^{d\times d^{\prime }}$ and $b_{G}\in R^{d^{\prime }}$ are the learnable weight and bias parameters.

The projected atomic features $\left(H_{i}^{\text{atomic\_proj}}\right)$ capture the structural composition of each motif. To enrich this with the motif’s explicit categorical identity, we combine it with the motif’s own dense embedding, $\text{E}\left(M_{i}\right)\in R^{n\times d^{\prime }}$ , which is obtained from a trainable embedding layer. The final fusion is achieved through element-wise addition:

$$H_{i}=H_{i}^{\text{atomic\_proj}}+\text{E}\left(M_{i}\right)$$ (9)

This additive fusion, illustrated in Figure 1b, ensures that the resulting representation for each motif is informed by both its constituent atoms and its learned semantic identity. During this process, the [GLOBAL] node’s embedding is temporarily set aside and subsequently reintroduced to yield the final fused feature matrix passed to the attention layers.

This multi-step fusion strategy is a deliberate design choice to create a more powerful representation than simpler alternatives. Unlike methods that might only use motif identities or perform a basic feature concatenation, our approach constructs a composite representation. The matrix multiplication $F_{i}X_{i}$ is not merely a feature-gathering step but a chemically-informed aggregation that respects the molecular structure. The subsequent additive fusion with identity embeddings is inspired by the success of residual connections in deep learning, allowing for the seamless integration of two complementary information sources. This ensures that the final motif representation is aware of both its internal atomic structure and its global categorical identity, leading to a more nuanced and effective input for the subsequent attention mechanism.

### 2.5 Global-local attention

The Transformer architecture, renowned for its self-attention mechanism, excels at modeling global dependencies and long-range interactions in sequential data. Its capacity for parallel computation and its multi-head design enable it to capture feature interactions across diverse representational subspaces. Inspired by these strengths, we propose a Global-Local Attention mechanism. This mechanism operates by partitioning the input feature dimension into two equal subspaces, dedicating one to a global attention module and the other to a local attention module. This dual-channel design allows the model to simultaneously track long-range topological relationships while precisely modeling fine-grained local structural variations.

The fused feature representation from the previous step is a matrix $H_{i}\in R^{N\times d}$ , where N is the number of motifs and d is the feature dimension. We partition this matrix along its feature dimension into two sub-matrices of equal size: $H_{g}\in R^{N\times \left(d/2\right)}$ and $H_{l}\in R^{N\times \left(d/2\right)}$ , as depicted in Figure 1c. The rationale for this partitioning is to create two distinct, non-overlapping channels for processing different types of structural information. We designate the first half, $H_{g}$ , as the input for the global channel, and the second half, $H_{l}$ , for the local channel. The global channel is augmented with the distance matrix $D\in R^{N\times N}$ to inform its attention calculation, while the local channel utilizes the adjacency matrix $A\in R^{N\times N}$ , as detailed in Figure 1d.

The global part is implemented as follows, where $W_{q}^{g},W_{k}^{g},W_{v}^{g}\in R^{\left(d/2\right)\times \left(d/2\right)}$ are learnable weight matrices:

$$Q_{g}=H_{g}W_{q}^{g},\;K_{g}=H_{g}W_{k}^{g},\;V_{g}=H_{g}W_{v}^{g},$$ (10)

$${\text{Attention}}_{g}=\text{softmax}\left(\frac{Q_{g}K_{g}^{⊤}⊙D}{\sqrt{\frac{d}{2}}}\right)V_{g}.$$ (11)

Similarly, the local part is computed as follows:

$$Q_{l}=H_{l}W_{q}^{l},\;K_{l}=H_{l}W_{k}^{l},\;V_{l}=H_{l}W_{v}^{l},$$ (12)

$${\text{Attention}}_{l}=\text{softmax}\left(\frac{Q_{l}K_{l}^{⊤}⊙A}{\sqrt{\frac{d}{2}}}\right)V_{l},$$ (13)

where $W_{q}^{l},W_{k}^{l},W_{v}^{l}\in R^{\left(d/2\right)\times \left(d/2\right)}$ are the corresponding learnable parameters for the local channel.

The complete attention block consists of an L -layer stacked architecture. The outputs from the global and local channels at each layer are concatenated along the feature dimension. To mitigate issues such as gradient vanishing, we incorporate residual connections and layer normalization. The final aggregated motif feature representation, $H^{\prime }$ , is thus obtained.

$$H^{\prime }=\text{LayerNorm}\left(\text{H}+\text{Concat}\left({\text{Attention}}_{g},{\text{Attention}}_{l}\right)\right).$$ (14)

Ultimately, this refined representation $H^{\prime }$ is integrated into the initial molecular representation via a learnable scaling factor α , yielding the final molecular representation $M^{\prime }=M+\alpha H^{\prime }$ . This comprehensive representation is then passed to a fully connected prediction layer to generate the final output for the prediction task.

## 3 Result and discussion

### 3.1 Datasets and training details

To comprehensively evaluate the performance of the DFusMol model, we selected nine benchmark datasets from diverse domains within MoleculeNet (Wu et al., 2018) for validation. As detailed in Table 1, these include the single-task classification datasets BACE and BBBP; the multi-task classification datasets SIDER, ClinTox, Tox21, and ToxCast; and the regression datasets ESOL, FreeSolv, and Lipophilicity.

**TABLE 1:** Statistics of the datasets used in the experiments. Dataset statistics include task numbers, types, molecule counts, and evaluation metrics.

Category	Dataset	Tasks	Task type	Molecules	Metric
Physiology	BBBP	1	Classification	2039	ROC-AUC
	Tox21	12	Classification	7,831	ROC-AUC
	ToxCast	617	Classification	8,575	ROC-AUC
	SIDER	27	Classification	1,427	ROC-AUC
	ClinTox	2	Classification	1,478	ROC-AUC
Biophysics	BACE	1	Classification	1,513	ROC-AUC
Physical chemistry	ESOL	1	Regression	1,128	RMSE
	FreeSolv	1	Regression	642	RMSE
	Lipophilicity	1	Regression	4,200	RMSE

^a^ Dataset statistics including task numbers, types, molecule counts, and evaluation metrics.

Following the recommended settings of MoleculeNet, the five classification datasets were evaluated using the average area under the receiver operating characteristic curve (ROC-AUC) as the metric. For the four regression tasks, the root mean square error (RMSE) was adopted to assess model performance.

All datasets were partitioned based on a scaffold-split methodology (Ramsundar et al., 2019), which provides a more rigorous test of a model’s generalization capabilities. A chemical scaffold is defined as the core molecular framework that remains after the removal of all substituents. During the dataset partitioning process, molecules are first clustered based on their chemical scaffolds, ensuring that molecules with identical scaffolds are grouped into the same subset. We prioritize the assignment of larger scaffold clusters to maintain a balanced distribution across the splits. Subsequently, the molecules are divided into training, validation, and test sets according to an 8:1:1 ratio.

Compared to random splitting, this scaffold-based strategy more accurately simulates real-world molecular prediction scenarios, particularly when the test set contains scaffolds not seen during training. This allows for a more robust evaluation of the model’s ability to generalize to novel chemical spaces. This approach yields more stable results, reduces performance fluctuations attributable to the partitioning method, and thereby enhances the reproducibility and generalizability of the study.

Distinct loss functions were employed to optimize for different task types. Specifically, classification tasks utilized the Binary Cross-Entropy with Logits Loss (BCEWithLogitsLoss), whereas regression tasks were optimized using the Mean Squared Error (MSE) loss function. We determined the optimal hyperparameters through a systematic search based on the model’s performance on the validation set. Key hyperparameters and their search spaces included: learning rates from 1e-3, 5e-4, 1e-4; batch sizes from 64, 128, 256; and the number of attention heads from 2, 4, 8. The configuration that yielded the best performance on the validation set was selected for the final model. This resulted in choosing the Adam optimizer with a learning rate of 1e-4 and a batch size of 256. The final model was then trained for 100 epochs.

In the network architecture, the input dimension of the attention mechanism was uniformly configured to 256. This was a deliberate choice to prevent potential errors related to dimensional partitioning during hyperparameter searches. To ensure the reliability of our experimental results, we conducted three independent trials for each experiment and reported the average performance on the test set. Classification tasks were evaluated using ROC-AUC, while regression tasks were assessed with RMSE. The model framework was implemented using the PyTorch library. All experiments were performed on a CentOS operating system equipped with an NVIDIA A100 GPU.

### 3.2 Comparative study on the MoleculeNet benchmark

We compared DFusMol with twelve baseline models, including supervised methods: GCN, GIN, MPNN, DMPNN, CMPNN, TransFoxMol, and pre-trained methods: GEM, GROVER, GraphMVP, MolCLR, KANO, and Unimol. Results for GROVER, GraphMVP, MolCLR, GEM, and KANO were sourced from the KANO paper (Fang et al., 2023). We also chose Unimol (Zhou et al., 2023) and TransFoxMol (Gao et al., 2023), both of which are built on Transformer models. We tested them using same training setups for a fair comparison. Since TansFoxMol reviews the dataset beforehand but the ToxCast data was not shared, there are no results to show in the table.

In comprehensive experiments on both classification (Table 2) and regression (Table 3) tasks, the non-pre-trained DFusMol model demonstrated outstanding predictive performance across a diverse range of molecular property prediction tasks. It achieved state-of-the-art (SOTA) results on four out of the six classification datasets. On the remaining two datasets, BACE and ToxCast, DFusMol performed slightly below the pre-trained KANO model, securing the second-best metrics.

**TABLE 2:** Test performance of thirteen models on six classification benchmarks of physiology and biophysics. The first five and the last two models are supervised learning methods, while the remaining six are self-supervised learning methods. The average and standard deviation of ROC-AUC (%) across three independent runs are reported (higher is better).

Dataset	BBBP	BACE	ClinTox	SIDER	Tox21	ToxCast
MPNN (Gilmer et al., 2020)	88.1 ± 4.0	82.6 ± 5.0	86.8 ± 3.0	59.4 ± 3.3	81.3 ± 2.1	67.7 ± 2.4
DMPNN (Gasteiger et al., 2020)	85.8 ± 5.6	76.8 ± 7.8	86.1 ± 2.3	58.4 ± 3.2	80.4 ± 2.2	66.7 ± 2.0
CMPNN (Song et al., 2020)	93.0 ± 2.4	86.7 ± 2.8	89.3 ± 4.3	60.2 ± 3.4	81.5 ± 1.8	69.3 ± 1.8
GCN (Kipf and Welling, 2016)	84.2 ± 3.7	75.2 ± 5.2	83.8 ± 2.4	52.8 ± 3.3	76.4 ± 1.9	60.9 ± 1.3
GIN (Xu et al., 2018)	85.5 ± 4.9	81.0 ± 5.2	86.9 ± 2.5	57.8 ± 3.9	79.2 ± 1.9	79.2 ± 1.9
GEM (Fang et al., 2022)	88.8 ± 0.4	87.9 ± 1.1	90.3 ± 0.7	63.2 ± 1.5	78.1 ± 0.4	68.6 ± 0.2
GROVE (Rong et al., 2020)	86.8 ± 2.2	82.4 ± 3.6	70.3 ± 13.7	61.2 ± 2.5	80.3 ± 2.0	56.8 ± 3.4
GraphMVP (Liu et al., 2021)	72.4 ± 1.6	81.2 ± 0.9	79.1 ± 2.8	63.9 ± 1.2	75.9 ± 0.5	63.1 ± 0.4
MolCLR (Wang et al., 2022)	73.3 ± 1.0	82.8 ± 0.7	89.8 ± 2.7	61.2 ± 3.6	74.1 ± 5.3	65.9 ± 2.1
KANO (Fang et al., 2023)	96.0 ± 1.6	**93.1** ± **2.1**	94.4 ± 0.3	65.2 ± 0.8	83.7 ± 1.3	**73.2** ± **1.6**
UniMol (Zhou et al., 2023)	90.3 ± 2.0	85.5 ± 3.2	86.6 ± 2.4	61.1 ± 1.6	83.6 ± 1.5	69.5 ± 2.4
TransFoxMol (Gao et al., 2023)	90.0 ± 2.8	81.0 ± 8.6	94.3 ± 9.5	61.4 ± 3.3	76.8 ± 1.6	–
DFusMol	**96.9** ± **0.3**	91.4 ± 0.7	**95.2** ± **0.1**	**65.7** ± **1.1**	**84.9** ± **0.6**	71.9 ± 1.1

The best results are marked in bold.

**TABLE 3:** Test performance of twelve models on three regression benchmarks in physical chemistry. The first five and the last two models are supervised learning methods, while the remaining five are self-supervised learning methods. The mean and standard deviation of test root mean square error (RMSE) over three independent runs are reported (lower is better).

Dataset	ESOL	FreeSolv	Lipophilicity
MPNN (Gilmer et al., 2020)	1.033 ± 0.109	1.879 ± 0.359	0.699 ± 0.083
DMPNN (Gasteiger et al., 2020)	1.117 ± 0.148	1.884 ± 0.354	0.736 ± 0.086
CMPNN (Song et al., 2020)	0.812 ± 0.104	1.679 ± 0.425	0.682 ± 0.070
GCN (Kipf and Welling, 2016)	1.439 ± 0.241	2.769 ± 0.550	0.913 ± 0.092
GIN (Xu et al., 2018)	1.378 ± 0.198	2.257 ± 0.510	0.825 ± 0.116
GEM (Fang et al., 2022)	0.813 ± 0.028	1.748 ± 0.114	0.674 ± 0.022
GROVER (Rong et al., 2020)	1.423 ± 0.288	2.940 ± 0.615	0.823 ± 0.010
MolCLR (Wang et al., 2022)	1.113 ± 0.023	2.301 ± 0.247	0.789 ± 0.009
KANO (Fang et al., 2023)	0.670 ± 0.019	1.142 ± 0.258	**0.566** ± **0.007**
UniMol (Zhou et al., 2023)	0.784 ± 0.082	1.735 ± 0.371	0.575 ± 0.045
TransFoxMol (Gao et al., 2023)	1.016 ± 0.059	2.341 ± 0.636	0.677 ± 0.019
DFusMol	**0.668** ± **0.005**	**1.081** ± **0.211**	0.576 ± 0.008

The best results are marked in bold.

An in-depth analysis of these results suggests potential reasons for KANO’s superior performance on the BACE and ToxCast datasets. The BACE dataset is known to be highly sensitive to molecular 3D conformations and the spatial arrangement of key functional groups. It is plausible that KANO’s pre-training regimen or its knowledge integration mechanism is better optimized for capturing such fine-grained structural details, thereby providing it with a competitive edge. The ToxCast dataset, which encompasses a large and complex set of toxicological endpoints, may benefit more significantly from extensive pre-training on diverse molecular data, a resource-intensive process.

Currently, the training configuration of DFusMol has not been specifically optimized for 3D conformational features or for large-scale, multi-task learning scenarios like those presented by ToxCast. Therefore, we believe there is potential for further performance improvements in these specific areas through targeted architectural or training enhancements.

In the regression tasks, DFusMol achieved the best results on the ESOL and FreeSolv datasets. On the Lipophilicity dataset, while its performance did not surpass that of KANO and UniMol, the performance gap was minimal, remaining within approximately 0.01 RMSE. This demonstrates the model’s high efficacy in predicting fundamental physicochemical properties.

Overall, DFusMol exhibited state-of-the-art or highly competitive performance across both classification and regression scenarios. This underscores its strong capability in modeling complex molecular structural features and its capacity for generalization across multiple tasks. For future work, we plan to investigate tailored training strategies for specific, challenging datasets such as BACE and ToxCast. Additionally, a deeper exploration of integrating 3D structural prior knowledge could further enhance the adaptability and performance of the DFusMol framework.

### 3.3 Ablation study

**FIGURE 3:**
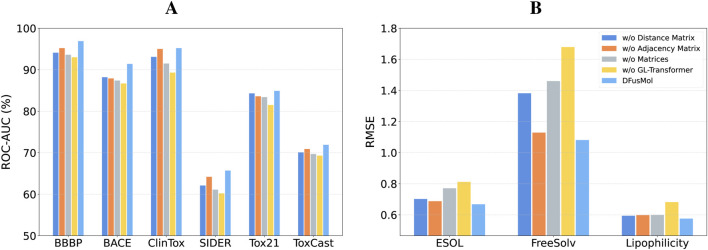
Impact of the adjacency matrix, distance matrix, and global-local attention module on performance across all tasks. **(A)** Classification tasks. **(B)** Regression tasks.

To validate the contribution of individual components within DFusMol, we conducted a series of ablation studies. These studies were designed to investigate the impact of the adjacency and distance matrices on the global-local attention mechanism, as well as the overall significance of the attention module itself. The results of these experiments are presented in Figure 3.

Our initial analysis focused on the attention module. A comparison between the “w/o GL-Transformer” and “w/o Matrices” model variants reveals an important finding. Although attention mechanisms can be sensitive to sample size, potentially leading to underfitting in low-data regimes, the fusion of motif-level and atomic-level features alone still yields competitive performance on datasets such as BACE, ClinTox, ESOL, and FreeSolv. This result underscores the criticality and effectiveness of our multi-scale feature fusion strategy. Specifically, the introduction of motif-level features not only enriches the model’s representation with higher-level structural information but also appears to partially compensate for the limitations of the attention mechanism in small-sample scenarios.

Next, we assessed the individual contributions of the auxiliary matrices by creating three distinct model variants: one that ablates the distance matrix (utilizing only the adjacency matrix for local attention), one that ablates the adjacency matrix (utilizing only the distance matrix for global attention), and a third that removes both auxiliary matrices entirely (‘w/o Matrices’). The results indicate that model performance exhibits differential sensitivity to the ablation of these matrices across datasets, a phenomenon we attribute to factors such as the task’s dependence on molecular structure, dataset size, and chemical property diversity. For tasks that are more dependent on local substructures or subtle topological differences—such as toxicity prediction (ClinTox/SIDER/Tox21/ToxCast) or blood-brain barrier permeability (BBBP)—the removal of the adjacency matrix results in a more pronounced performance drop. In contrast, on the BACE dataset, which requires higher fidelity to 3D conformations and global physicochemical properties, performance degrades more significantly in the absence of the distance matrix. When both auxiliary matrices are ablated, the model’s predictive capability is substantially diminished.

**FIGURE 4:**
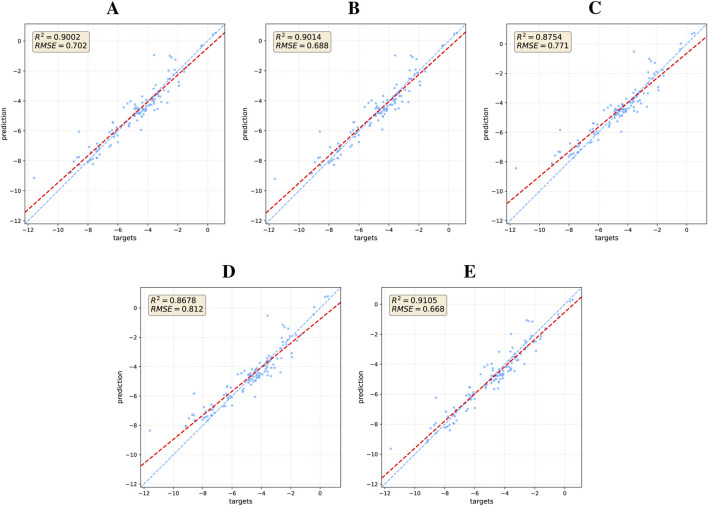
Regression performance of DFusMol and its ablated versions on the ESOL dataset. The blue dashed line indicates the line of perfect correlation (y = x), while the red dashed line represents the linear best fit to the data, indicating the model’s actual predictive trend: **(A)** DFusMol without Distance Matrix, **(B)** DFusMol without Adjacency Matrix, **(C)** DFusMol without Matrix inputs, **(D)** DFusMol without GL-Transformer, and **(E)** DFusMol.

**FIGURE 5:**
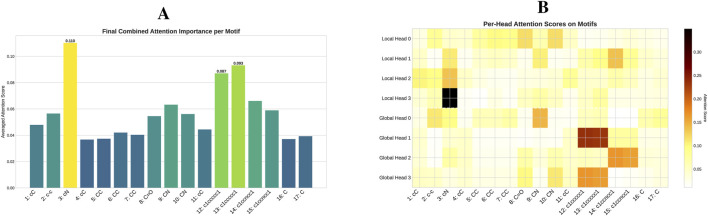
Attention analysis of a selected molecule from the BACE test set. The figure details the attention scores from the final layer of the model for a specific molecule (O=C(NCc1cccnc1)C(Cc1cc2cc (ccc2nc1N)-c1ccccc1C) **(C)** with a positive label (target = 1). **(A)** Average attention scores of the GLOBAL node on other motifs, computed separately for the four Local heads and four Global heads. **(B)** The heatmap displaying the per-head attention scores of the GLOBAL node on other motifs, providing a granular view of how each individual head distributes its attention across the motifs.

The quantitative impact of each model component is visually demonstrated through ablation studies on the ESOL dataset (Figure 4). These results reaffirm that a comprehensive integration of local and global structural information is critical for enhancing model performance. To understand the mechanism behind these gains, particularly from the Transformer, we delved deeper by analyzing its attention patterns. In a case study on a representative molecule from the BACE test set (target = 1), we visualized the attention distribution from the final layer, as detailed in the heatmap in Figure 5. This analysis reveals a crucial insight: the Transformer directs a high degree of attention towards critical motifs. This finding provides a compelling rationale for the quantitative results, confirming that the Transformer’s effectiveness stems from its ability to identify and leverage key chemical features for property prediction.

## 4 Conclusion

In this work, we introduced DFusMol, a novel framework for molecular representation learning that integrates information from both molecular graphs and motif-level graphs. By leveraging auxiliary information from adjacency and distance matrices, DFusMol captures a more comprehensive structural representation of molecules across multiple scales, thereby enhancing the accuracy of property prediction. A key innovation of our approach is the dual-channel attention mechanism, which not only effectively fuses atomic-level and motif-level information but also provides a transparent framework for interpreting the model’s decision-making process. Our extensive evaluations on the MoleculeNet benchmark demonstrate that DFusMol achieves state-of-the-art or highly competitive performance across a diverse set of classification and regression tasks. Furthermore, ablation studies confirm that the integration of multi-level structural information provides a substantial performance enhancement. Our analysis of the attention mechanism further reveals that the model learns to focus on chemically relevant motifs, offering a clear rationale for its strong predictive power. Despite its strong performance, DFusMol does not consistently surpass specialized, pre-trained models on tasks that are highly dependent on 3D molecular conformation. Future work will therefore focus on three primary avenues: enhancing the current framework, expanding its applications, and broadening its evaluation. To enhance the model, we will focus on incorporating 3D structural priors and leveraging larger-scale pre-training. To expand its scope, we will explore its utility for other critical tasks, such as *de novo* molecule generation and drug-target interaction (DTI) prediction. Finally, to more rigorously assess the model’s generalization and practical relevance, we plan to benchmark DFusMol on datasets beyond MoleculeNet, such as those from quantum mechanics (e.g., QM9) and carefully-curated drug discovery challenges. These directions present new challenges but also offer significant opportunities to improve the adaptability and performance of the DFusMol framework. In summary, DFusMol provides a new perspective on molecular modeling that extends beyond atom-centric representations, delivering a framework that excels in both predictive accuracy and mechanistic interpretability. We believe this framework holds considerable potential for future evolution and can serve as a powerful and interpretable tool for drug discovery and molecular science, stimulating further research and practical applications in these fields.

## Data Availability

Publicly available datasets were analyzed in this study. This data can be found here: https://github.com/Fireflower-March/DFusMol.

## Abbreviations

SVM, Support Vector Machines; RF, Random Forest; GNN, Graph Neural Network; MPNN, Message Passing Neural Network; DMPNN, Directed Message Passing Neural Network; CMPNN, Communicative Message Passing Neural Network; GAT, Graph Attention Network; GIN, Graph Isomorphism Network; GCN, Graph Convolutional Network; GRU, Gated Recurrent Unit; ROC, Receiver Operating Characteristic; MSE, Mean Squared Error; RMSE, Root Mean Squared Error; AUC, Area Under the Curve; BCE, Binary Cross-Entropy; w/o, Without.

## References

[B1] Ahneman D. T. Estrada J. G. Lin S. Dreher S. D. Doyle A. G. (2018). Predicting reaction performance in c–n cross-coupling using machine learning. Science 360, 186–190. 10.1126/science.aar5169 29449509

[B2] Ashish V. (2017). Attention is all you need. Adv. neural Inf. Process. Syst. 30 (I). 10.48550/arXiv.1706.03762

[B3] Boser B. E. Guyon I. M. Vapnik V. N. (1992). “A training algorithm for optimal margin classifiers,” in Proceedings of the fifth annual workshop on Computational learning theory, 144–152.

[B4] Breiman L. (2001). Random forests. Mach. Learn. 45, 5–32. 10.1023/a:1010933404324

[B5] Cho K. Van Merriënboer B. Bahdanau D. Bengio Y. (2014). On the properties of neural machine translation: encoder-decoder approaches. arXiv Prepr. arXiv:1409). 10.48550/arXiv.1409.1259

[B6] Deng J. Yang Z. Wang H. Ojima I. Samaras D. Wang F. (2023). A systematic study of key elements underlying molecular property prediction. Nat. Commun. 14, 6395. 10.1038/s41467-023-41948-6 37833262 PMC10575948

[B7] Dowden H. Munro J. (2019). Trends in clinical success rates and therapeutic focus. Nat. Rev. Drug Discov. 18, 495–496. 10.1038/d41573-019-00074-z 31267067

[B8] Fang X. Liu L. Lei J. He D. Zhang S. Zhou J. (2022). Geometry-enhanced molecular representation learning for property prediction. Nat. Mach. Intell. 4, 127–134. 10.1038/s42256-021-00438-4

[B9] Fang Y. Zhang Q. Zhang N. Chen Z. Zhuang X. Shao X. (2023). Knowledge graph-enhanced molecular contrastive learning with functional prompt. Nat. Mach. Intell. 5, 542–553. 10.1038/s42256-023-00654-0

[B10] Fedik N. Zubatyuk R. Kulichenko M. Lubbers N. Smith J. S. Nebgen B. (2022). Extending machine learning beyond interatomic potentials for predicting molecular properties. Nat. Rev. Chem. 6, 653–672. 10.1038/s41570-022-00416-3 37117713

[B11] Gao J. Shen Z. Xie Y. Lu J. Lu Y. Chen S. (2023). Transfoxmol: predicting molecular property with focused attention. Briefings Bioinforma. 24, bbad306. 10.1093/bib/bbad306 37605947

[B12] Gasteiger J. Groß J. Günnemann S. (2020). Directional message passing for molecular graphs. arXiv Prepr. arXiv:2003.03123. 10.48550/arXiv.2003.03123

[B13] Gaulton A. Bellis L. J. Bento A. P. Chambers J. Davies M. Hersey A. (2012). Chembl: a large-scale bioactivity database for drug discovery. Nucleic acids Res. 40, D1100–D1107. 10.1093/nar/gkr777 21948594 PMC3245175

[B14] Gilmer J. Schoenholz S. S. Riley P. F. Vinyals O. Dahl G. E. (2020). “Message passing neural networks,” in Machine learning meets quantum physics (Springer), 199–214.

[B15] Guo Z. Guo K. Nan B. Tian Y. Iyer R. G. Ma Y. (2022). Graph-based molecular representation learning. arXiv Prepr. arXiv:2207.04869. 10.48550/arXiv.2207.04869

[B16] Hall L. H. Mohney B. Kier L. B. (1991). The electrotopological state: structure information at the atomic level for molecular graphs. J. Chem. Inf. Comput. Sci. 31, 76–82. 10.1021/ci00001a012

[B17] Harnik Y. Milo A. (2024). A focus on molecular representation learning for the prediction of chemical properties. Chem. Sci. 15, 5052–5055. 10.1039/d4sc90043j 38577350 PMC10988574

[B18] Hay M. Thomas D. W. Craighead J. L. Economides C. Rosenthal J. (2014). Clinical development success rates for investigational drugs. Nat. Biotechnol. 32, 40–51. 10.1038/nbt.2786 24406927

[B19] Hou Y. Wang S. Bai B. Chan H. S. Yuan S. (2022). Accurate physical property predictions via deep learning. Molecules 27, 1668. 10.3390/molecules27051668 35268770 PMC8912091

[B20] Keiser M. J. Setola V. Irwin J. J. Laggner C. Abbas A. I. Hufeisen S. J. (2009). Predicting new molecular targets for known drugs. Nature 462, 175–181. 10.1038/nature08506 19881490 PMC2784146

[B21] Kipf T. N. Welling M. (2016). Semi-supervised classification with graph convolutional networks. arXiv Prepr. arXiv:1609.02907. 10.48550/arXiv.1609.02907

[B22] Landrum G. (2016). Rdkit: open-source cheminformatics software. Available online at: https://github.com/rdkit/rdkit/releases/tag/Release_2016_09_4.

[B23] Li Y. Hsieh C.-Y. Lu R. Gong X. Wang X. Li P. (2022). An adaptive graph learning method for automated molecular interactions and properties predictions. Nat. Mach. Intell. 4, 645–651. 10.1038/s42256-022-00501-8

[B24] Liu S. Wang H. Liu W. Lasenby J. Guo H. Tang J. (2021). Motif-based graph self-supervised learning for molecular property prediction. arXiv Prepr. arXiv:2110.07728. 10.48550/arXiv.2110.00987

[B25] Ni Y. Feng S. Hong X. Sun Y. Ma W.-Y. Ma Z.-M. (2024). Pre-training with fractional denoising to enhance molecular property prediction. Nat. Mach. Intell. 6, 1169–1178. 10.1038/s42256-024-00900-z

[B26] Ramsundar B. Eastman P. Walters P. Pande V. (2019). MoleculeNet: a benchmark for molecular machine learning. Deep Learn. life Sci. Appl. deep Learn. genomics, Microsc. drug Discov. more (O’Reilly Media).

[B27] Rong Y. Bian Y. Xu T. Xie W. Wei Y. Huang W. (2020). Self-supervised graph transformer on large-scale molecular data. Adv. Neural. Inf. Process Syst. 33, 12559–12571.

[B28] Ross J. Belgodere B. Chenthamarakshan V. Padhi I. Mroueh Y. Das P. (2022). Large-scale chemical language representations capture molecular structure and properties. Nat. Mach. Intell. 4, 1256–1264. 10.1038/s42256-022-00580-7

[B29] Sadybekov A. V. Katritch V. (2023). Computational approaches streamlining drug discovery. Nature 616, 673–685. 10.1038/s41586-023-05905-z 37100941

[B30] Schwaller P. Laino T. Gaudin T. Bolgar P. Hunter C. A. Bekas C. (2019). Molecular transformer: a model for uncertainty-calibrated chemical reaction prediction. ACS central Sci. 5, 1572–1583. 10.1021/acscentsci.9b00576 PMC6764164 31572784

[B31] Song Y. Zheng S. Niu Z. Fu Z. H. Lu Y. Yang Y. (2020). “Communicative representation learning on attributed molecular graphs,” in Proceedings of the Twenty-Ninth International Joint Conference on Artificial Intelligence, IJCAI-20 2831-2838. Editor C. Bessiere. International Joint Conferences on Artificial Intelligence Organization, 2831–2838. 10.24963/ijcai.2020/392

[B32] Sultan A. Sieg J. Mathea M. Volkamer A. (2024). Transformers for molecular property prediction: lessons learned from the past five years. J. Chem. Inf. Model. 64, 6259–6280. 10.1021/acs.jcim.4c00747 39136669

[B33] Veličković P. Cucurull G. Casanova A. Romero A. Lio P. Bengio Y. (2017). Graph attention networks. arXiv Prepr. arXiv:1710.10903. 10.48550/arXiv.1710.10903

[B34] Wang Y. Wang J. Cao Z. Barati Farimani A. (2022). Molecular contrastive learning of representations via graph neural networks. Nat. Mach. Intell. 4, 279–287. 10.1038/s42256-022-00447-x

[B35] Wei Z.-S. Han K. Yang J.-Y. Shen H.-B. Yu D.-J. (2016). Protein–protein interaction sites prediction by ensembling svm and sample-weighted random forests. Neurocomputing 193, 201–212. 10.1016/j.neucom.2016.02.022

[B36] Weininger D. Weininger A. Weininger J. L. (1989). Smiles. 2. algorithm for generation of unique smiles notation. J. Chem. Inf. Comput. Sci. 29, 97–101. 10.1021/ci00062a008

[B37] Wu Z. Ramsundar B. Feinberg E. N. Gomes J. Geniesse C. Pappu A. S. (2018). Moleculenet: a benchmark for molecular machine learning. Chem. Sci. 9, 513–530. 10.1039/c7sc02664a 29629118 PMC5868307

[B38] Xu K. Hu W. Leskovec J. Jegelka S. (2018). How powerful are graph neural networks? arXiv Prepr. arXiv:1810, 00826. 10.48550/arXiv.1810.00826

[B39] Yang H. Xiu J. Yan W. Liu K. Cui H. Wang Z. (2025). Large language models as tools for molecular toxicity prediction: ai insights into cardiotoxicity. J. Chem. Inf. Model. 65, 2268–2282. 10.1021/acs.jcim.4c01371 39982968

[B40] Yu Z. Gao H. (2022). “Molecular representation learning via heterogeneous motif graph neural networks,” in International conference on machine learning (PMLR), 25581–25594.

[B41] Zeng X. Xiang H. Yu L. Wang J. Li K. Nussinov R. (2022). Accurate prediction of molecular properties and drug targets using a self-supervised image representation learning framework. Nat. Mach. Intell. 4, 1004–1016. 10.1038/s42256-022-00557-6

[B42] Zhang J. Du W. Yang X. Wu D. Li J. Wang K. (2023). Smg-bert: integrating stereoscopic information and chemical representation for molecular property prediction. Front. Mol. Biosci. 10, 1216765. 10.3389/fmolb.2023.1216765 37457837 PMC10348360

[B43] Zhang X. Chen C. Wang X. Jiang H. Zhao W. Cui X. (2025). Metagin: a lightweight framework for molecular property prediction. Front. Comput. Sci. 19, 195912. 10.1007/s11704-024-3784-y

[B44] Zhang Z. Liu Q. Wang H. Lu C. Lee C.-K. (2021). Motif-based graph self-supervised learning for molecular property prediction. Adv. Neural Inf. Process. Syst. 34, 15870–15882.

[B45] Zhong Y. Li G. Yang J. Zheng H. Yu Y. Zhang J. (2024). Learning motif-based graphs for drug–drug interaction prediction via local–global self-attention. Nat. Mach. Intell. 6, 1094–1105. 10.1038/s42256-024-00888-6

[B46] Zhou G. Gao Z. Ding Q. Zheng H. Xu H. Wei Z. (2023). Uni-mol: a universal 3d molecular representation learning framework

